# The Technical Efficiency of Earthquake Medical Rapid Response Teams Following Disasters: The Case of the 2010 Yushu Earthquake in China

**DOI:** 10.3390/ijerph121214991

**Published:** 2015-12-04

**Authors:** Xu Liu, Bihan Tang, Hongyang Yang, Yuan Liu, Chen Xue, Lulu Zhang

**Affiliations:** 1 Department of Military Health Management, College of Military Health Management, Second Military Medical University, Shanghai 200433, China; aqualau@126.com (X.L.); mangotangbihan@126.com (B.T.); yawnlau@126.com (Y.L.); xue1990chen@163.com (C.X.); 2 Department of Medical Affairs, Second Artillery General Hospital of Chinese People’s Liberation Army, Beijing 100088, China; yanghy1986@163.com

**Keywords:** earthquake medical response team, technical efficiency, data envelopment analysis, tobit regression, Yushu earthquake

## Abstract

*Purpose*: Performance assessments of earthquake medical rapid response teams (EMRRTs), particularly the first responders deployed to the hardest hit areas following major earthquakes, should consider efficient and effective use of resources. This study assesses the daily technical efficiency of EMRRTs in the emergency period immediately following the 2010 Yushu earthquake in China. *Methods*: Data on EMRRTs were obtained from official daily reports of the general headquarters for Yushu earthquake relief, the emergency office of the National Ministry of Health, and the Health Department of Qinghai Province, for a sample of data on 15 EMRRTs over 62 days. Data envelopment analysis was used to examine the technical efficiency in a constant returns to scale model, a variable returns to scale model, and the scale efficiency of EMRRTs. Tobit regression was applied to analyze the effects of corresponding influencing factors. *Results*: The average technical efficiency scores under constant returns to scale, variable returns to scale, and the scale efficiency scores of the 62 units of analysis were 77.95%, 89.00%, and 87.47%, respectively. The staff-to-bed ratio was significantly related to global technical efficiency. The date of rescue was significantly related to pure technical efficiency. The type of institution to which an EMRRT belonged and the staff-to-bed ratio were significantly related to scale efficiency. *Conclusions*: This study provides evidence that supports improvements to EMRRT efficiency and serves as a reference for earthquake emergency medical rapid assistance leaders and teams.

## 1. Introduction

China is one of the most earthquake-prone countries in the world and the scale and response speed of China’s medical rescue teams for earthquake relief operations have attracted worldwide attention [1,2]. On 12 May 2008, the major Wenchuan earthquake, registering 8.0 on the Richter scale, hit Sichuan Province, China, resulting in more than 69,000 deaths. In addition to the almost 100,000 medical professionals working in Sichuan Province, a large number of supporting medical personnel, totaling about 10,630 medical professionals from across China, 7061 military health officers, and more than 350 international first-aid workers from 10 countries, rushed to the earthquake site [3]. Almost exactly two years later, at 7:49 am on 14 April 2010, another catastrophic earthquake, registering 7.1 on the Richter scale, hit Yushu, China, causing 2698 deaths and 12,135 injuries. Although the rescue effort was smaller than for the Wenchuan disaster, the government and military organized many earthquake medical rapid response teams (EMRRTs). These teams, together with EMRRTs from non-governmental and volunteer organizations, participated in the Yushu rescue efforts [4].

Some studies that have assessed the effectiveness of these efforts have found that wasted resources and inefficiency in the process of disaster relief should be issues of concern [3,5,6], but no studies have been conducted that specifically assess the efficiency of earthquake medical assistance. Our previous study found that assessing the efficiency of earthquake medical response should be the next research priority in this area of study, for China and the international community [7]. Thus, the primary goal of this study is to address this gap in the literature by analyzing the efficiency of disaster medical assistance by EMRRTs during the Yushu earthquake.

Data envelopment analysis (DEA) is a linear regression tool used to evaluate the relative effectiveness of types of organizational performance. DEA is particularly appropriate for evaluating the efficiency of health services’ delivery, which typically is a complex and multidimensional process, and it has proven to be an effective and versatile tool for healthcare efficiency measurement. DEA has been effectively employed to evaluate the technical efficiency and productivity of public hospitals [8], the supply of particular medical projects [9], and the effects of new techniques on the efficiency of medical institutions [10]. Currently, DEA and stochastic frontier analysis (SFA) are the most common methods used for these types of analyses [11]. Both of them can identify the production frontier of a group of facilities, but they employ different assumptions and methodologies. DEA methods use mathematical computations to obtain the production frontier enveloping all the observed data. Its main advantages are flexibility and versatility; moreover, it requires no information on relative prices and it easily accommodates multiple inputs and outputs. It is also computationally straightforward [12].

This study used the DEA method to examine the technical efficiency of EMRRTs after the 2010 Yushu earthquake. In addition, Tobit regression analysis was used to analyze the effects of relevant influencing factors. The study’s central goal was to obtain evidence to support improvements to EMRRT efficiency and a reference for emergency medical assistance professionals in earthquake relief.

## 2. Methods and Materials

### 2.1. Data Sources

The data were obtained from official daily reports of the general headquarters for Yushu earthquake relief, the emergency office of the National Ministry of Health, and the Health Department of Qinghai Province. During the first two weeks after the Yushu earthquake, the general headquarters of Yushu earthquake relief was the repository for all first-hand institutional rescue data from the hardest hit areas. These data included information on the rescue institutions’ allocations, human resources, workload, and the other information that was recorded in the official daily reports. Supported by the Emergency Response Office at the National Ministry of Health, the emergency medical rescue system of Yushu earthquake relief data were summarized and assessed from October of 2010 through October of 2011. Additional data were collected in a survey of closed-ended questions disseminated by e-mail, post, and in face-to-face interviews.

### 2.2. Analytical Methodology

First, DEA methods were used to assess the daily technical efficiency of each EMRRT (*n* = 15) during the initial emergency period after the Yushu earthquake. A medical institution is considered technically efficient when it produces the maximum output from a given amount of input or, alternatively, produces a given output with minimum inputs. Thus, when a medical institution is technically efficient, it operates on its production frontier [13].

The DEA methods use mathematical calculations to obtain the production frontier that envelops all of the observed data. The scores reflect each EMRRT’s performance relative to the best performers. In previous studies on earthquake relief, the initial emergency period was defined as lasting between 24 h and one week after the earthquake hit [14]. In the Yushu earthquake, 12,128 people were known injured by the fourth day after the earthquake hit (18 April 2010), which accounted for 99.94% of all the injured people, suggesting that the vast majority of rescue work was accomplished within the first four days [15]. Therefore, this study defined the emergency period of this particular earthquake as beginning on the day of the earthquake (14 April) and ending on the fourth day after the earthquake (18 April), for a total of five days.

The second analytical method used was Tobit regression, which estimated (maximum likelihood estimate) the effects of relevant variables on EMRRT efficiency. This approach was preferred to linear regression (OLS) because DEA efficiency scores are continuous from zero to one where the value of one is positive probability and the probability of obtaining the limiting value of zero is zero.

### 2.3. Sample Selection

The urgency that characterizes emergency medical rescue during earthquake relief influences the quality of the data recorded in the field by the medical rescue team members, and it often has missing or inconsistent entries. Field data differ from the data collected by health service institutions under normal conditions in that these data problems are intrinsic to the emergency, crisis context of the first days after a disaster.

For EMRRTs to be eligible for the study, they had to meet the following criteria. First, they were first responder teams performing triage, first aid, resuscitation, emergency care, and preparations for evacuation during the first five days following the earthquake; Second, they were deployed to the hardest hit areas; Third, they were proximate to other teams. Last, they provided data on all of the variables analyzed in the study. In this study, one day was defined as the unit of analysis. For example, Team X was involved in the rescue task for five days, which provided five days of data from X. Fifteen EMRRTs were deployed to the hardest hit areas and were proximate to each other, and rescue data on 62 of the 75 theoretical days were analyzed.

### 2.4. Variable Selection

Table 1 lists the variables used in the analysis. The number of doctors, the number of nurses, and the number of sickbeds were the input variables. The number of injured people who received treatment and the number of patients treated for medical problems not related to the earthquake were the output variables. Based on the effects of the input on the output variables, DEA methods assessed the daily technical efficiency of each of the 15 EMRRTs during the five-day emergency period. The rescue date, the EMRRT’s region of origin, the type of institution to which each EMRRT belonged, the staff-to-bed ratio, and the doctor-to-nurse ratio were included as variables of possible influence on the output variables. According to Chinese hospitals’ classification standards, hospitals are classified into one of three tiers based on functions, facilities, and extent or level of technological advancement.

- Primary hospitals have 100 or fewer beds, directly provide the local communities with medical care, preventive care, and other integrated services, and are the primary health care institutions.
- Secondary hospitals have between 101 and 500 beds and are regional hospitals that provide comprehensive healthcare services to multiple communities.
- Tertiary hospitals have more than 500 beds; provide hospital and other health services at the municipal, regional, provincial, and national levels; and are medical prevention and technology centers with the ability to conduct comprehensive healthcare, teaching, and research activities.

**Table 1:** List of variables used in the analysis with their definitions.

Variables		Definition
Input variables	Doctors	Number of doctors in each EMRRT
	Nurses	Number of nurses in each EMRRT
	Sickbeds	Number of sickbeds in each EMRRT
Output variables	Number of injured patients	Number of people receiving treatment for injuries sustained in the earthquake daily
	Number of non-injured patients	Number of people treated daily for other (non-earthquake) medical problems
Control Variables	Date	Rescue date
		0 = 4/14, 1 = 4/15, 2 = 4/16, 3 = 4/17, 4 = 4/18
	Area	EMRRT’s region of origin
		1 = Yushu, 2 = Regions in Qinghai Province, 3 = Regions outside of Qinghai Province
	Source	Type of institution to which each EMRRT belonged
		1 = primary hospital, 2 = secondary hospital, 3 = tertiary hospital
	Staff-to-bed ratio	Staff-to-bed ratio of each EMRRT
	Doctor-to-nurse ratio	Doctor-to-nurse ratio of each EMRRT

### 2.5. DEA Model Orientation

Technical efficiency measures the ability of a decision-making unit (DMU) to produce the maximum number of program outputs from a given number of inputs or a minimum input level set at a given number of outputs. In emergency medical rescue efforts in earthquake relief, which are based on a humanitarian spirit and the principle of saving lives, EMRRTs’ primary goal is to speedily rescue as many sick and injured people as possible. Therefore, this study chose the output-oriented mode. In this study, there were 62 DMUs, representing the 62 days of coverage by the 15 EMRRTs in the first five days after the earthquake.

Charnes [16] proposed a DEsA model that assumes constant returns to scale (CRS). However, the CRS assumption is appropriate only when all EMRRTs are operating at optimal level. Banker [17] subsequently suggested an extension of the CRS model to account for situations of variable returns to scale (VRS). The VRS model allows the best practice level of outputs to inputs to vary by the size of the facilities assessed, whereas CRS uses the highest achievable ratio of outputs to inputs of each unit (DMU) regardless of size.

Therefore, technical efficiency can be decomposed into pure technical efficiency (PTE) and scale efficiency (SE) by modeling both on the same data. If the results were to find the VRS and CRS have different technical efficiency (TE) scores on a given EMRRT, that EMRRT has demonstrated scale inefficiency, which can be calculated by taking the difference between the VRS TE score and the CRS TE score. CRS technical efficiency (CRSTE) is global TE, indicated by the TE scores under the CRS model. The VRS technical efficiency (VRSTE) is PTE, indicated by the TE scores under VRS model. Computing the ratio of CRSTE to VRSTE derives the SE. Scale inefficiency, present when SE < 100%, occurs when the facility (EMRRT) is not operating at its most productive (optimal) level [18]. In this study, the CRS and VRS models were both computed to obtain these three scores.

### 2.6. Data Analysis

Data Envelopment Analysis Program (DEAP) 2.1 was used to assess the efficiencies of 62 days of data of 15 EMRRTs (62 DMUs). Tobit regression was employed to examine the effects of control variables expected to be associated with the DEA scores using Stata Version 12.0. A value of *p* < 0.05 was the cut-off level of statistical significance.

## 3. Results

### 3.1. Descriptive Statistics and Analysis

Table 2 shows the descriptive statistics of the 62 DMUs with respect to all of the variables used in the analysis. There were 15 local DMUs (24.19%) from Yushu Tibetan Autonomous Prefecture, 34 DMUs (54.84%) from Qinghai Province, and 13 DMUs (20.97%) from outside Qinghai Province. There were 19 DMUs (30.65%) from primary hospitals, 17 DMUs (27.42%) from secondary hospitals, and 26 DMUs (41.94%) from tertiary hospitals. The average numbers of doctors, nurses, and sickbeds were 8.54 (*SD* = 5.22), 4.77 (*SD* = 3.97), and 6.00 (*SD* = 4.81), respectively. The staff-to-bed ratio and the doctor-to-nurse ratio ranged from 1.38:1 to 3.5:1 and from 0.75:1 to 6.0:1, respectively. The average numbers of injured people that were treated and the average numbers of people treated for non-earthquake medical problems were 41.52 (*SD* = 36.59) and 21.71 (*SD* = 18.71), respectively.

**Table 2:** Descriptive statistics of the 62 DMUs.

DMU	Date	Area	Source	S-B Ratio	D-N Ratio	Doctors	Nurses	Sickbeds	NIP	NNIP
A-1	4/15	2	3	3.00	2.00	8	4	4	112	4
A-2	4/16	2	3	3.00	2.00	8	4	4	89	21
A-3	4/17	2	3	3.00	2.00	8	4	4	22	38
A-4	4/18	2	3	3.00	2.00	8	4	4	18	40
B-2	4/16	2	2	3.40	2.40	12	5	5	77	42
B-3	4/17	2	2	3.33	1.86	13	7	6	39	51
B-4	4/18	2	2	3.33	1.86	13	7	6	13	57
C-0	4/14	2	2	2.25	0.80	4	5	4	102	0
C-1	4/15	2	2	2.25	0.80	4	5	4	93	0
C-2	4/16	2	2	2.25	0.80	4	5	4	32	26
C-3	4/17	2	2	2.25	0.80	4	5	4	13	35
C-4	4/18	2	2	2.25	0.80	4	5	4	8	31
D-1	4/15	2	2	2.33	0.75	6	8	6	78	22
D-2	4/16	2	2	2.33	0.75	6	8	6	59	28
D-3	4/17	2	2	2.33	0.75	6	8	6	27	31
D-4	4/18	2	2	2.33	0.75	6	8	6	14	32
E-0	4/14	2	2	3.50	1.33	4	3	2	74	3
E-1	4/15	2	2	3.50	1.33	4	3	2	61	5
E-2	4/16	2	2	3.50	1.33	4	3	2	30	10
E-3	4/17	2	2	3.50	1.33	4	3	2	12	18
E-4	4/18	2	2	3.50	1.33	4	3	2	5	29
F-0	4/14	2	3	2.75	4.50	9	2	4	78	20
F-1	4/15	2	3	2.75	4.50	9	2	4	76	18
F-2	4/16	2	3	2.75	4.50	9	2	4	52	22
F-3	4/17	2	3	2.75	4.50	9	2	4	21	35
F-4	4/18	2	3	2.75	4.50	9	2	4	9	28
G-0	4/14	1	1	2.00	3.00	15	5	10	117	9
G-1	4/15	1	1	2.00	3.00	15	5	10	78	36
G-2	4/16	1	1	2.00	3.00	15	5	10	31	33
G-3	4/17	1	1	2.00	3.00	15	5	10	27	61
G-4	4/18	1	1	2.00	3.00	15	5	10	14	62
H-0	4/14	1	1	3.00	5.00	10	2	4	81	5
H-1	4/15	1	1	3.00	5.00	10	2	4	60	22
H-2	4/16	1	1	3.00	5.00	10	2	4	24	33
H-3	4/17	1	1	3.00	5.00	10	2	4	13	27
H-4	4/18	1	1	3.00	5.00	10	2	4	7	29
I-0	4/14	1	1	2.33	6.00	6	1	3	76	9
I-1	4/15	1	1	2.33	6.00	6	1	3	17	22
I-2	4/16	1	1	1.67	4.00	4	1	3	5	17
I-3	4/17	1	1	1.67	4.00	4	1	3	4	10
I-4	4/18	1	1	1.67	4.00	4	1	3	4	9
J-2	4/16	3	3	3.00	1.00	3	3	2	32	18
J-3	4/17	3	3	3.00	1.00	3	3	2	11	21
J-4	4/18	3	3	1.50	2.00	2	1	2	5	12
K-2	4/16	3	3	2.00	1.00	4	4	4	34	18
K-3	4/17	3	3	2.00	1.00	4	4	4	14	20
K-4	4/18	3	3	2.00	1.00	4	4	4	10	23
L-1	4/15	3	1	3.00	2.00	2	1	1	23	10
L-2	4/16	3	1	3.00	2.00	2	1	1	13	12
L-3	4/17	3	1	3.00	2.00	2	1	1	7	10
L-4	4/18	3	1	3.00	2.00	2	1	1	6	6
M-2	4/16	2	3	1.38	4.50	18	4	16	76	21
M-3	4/17	2	3	1.38	4.50	18	4	16	42	25
M-4	4/18	2	3	1.50	5.00	20	4	16	14	41
N-0	4/14	2	3	2.20	1.20	12	10	10	152	27
N-1	4/15	2	3	2.20	1.20	12	10	10	134	49
N-2	4/16	2	3	2.20	1.20	12	10	10	67	44
N-3	4/17	2	3	2.20	1.20	12	10	10	23	76
N-4	4/18	2	3	2.20	1.20	12	10	10	15	77
0-2	4/16	3	3	1.85	1.06	19	18	20	76	52
0-3	4/17	3	3	1.90	1.11	20	18	20	38	51
0-4	4/18	3	3	1.90	1.11	20	18	20	10	75

Abbreviations: S-B Ratio = staff-to-bed ratio; D-N Ratio = doctor-to-nurse ratio; NIP = number of people receiving treatment daily for injuries sustained in the earthquake; NNIP = number of people treated daily for other (non-earthquake) medical problems daily.

### 3.2. Technical Efficiency Scores

Table 3 shows the technical efficiency scores by rescue date, the EMRRT’s region of origin, and the type of institution to which each EMRRT belonged. The average CRSTE, VRSTE, and SE scores of the total 62 DMUs were 77.95% (*SD* = 0.18), 89.00% (*SD* = 0.14), and 87.47% (*SD* = 0.15), respectively. The CRSTE scores of 12 units (19.35%) reached 100%, the VRSTE scores of 24 units (38.71%) reached 100%, and the SE of 21 units (33.87%) reached 100%.

**Table 3:** Summary of DEA scores by date, area, and source.

Statistic	Value of Variables	CRSTE			VRSTE			Scale Efficiency		
		Mean (%)	SD	100% TE	Mean (%)	SD	100% TE	Mean (%)	SD	100% TE
Date	0	59.9	0.154	8.70%	99.5	0.002	17.39%	60.15	0.153	8.70%
	1	87.27	0.022	0.00%	95.47	0.041	33.33%	91.62	0.057	16.67%
	2	73.03	0.192	9.09%	87.97	0.11	27.27%	82.57	0.169	9.09%
	3	72.99	0.22	18.18%	85.89	0.145	27.27%	83.91	0.176	18.18%
	4	77.07	0.172	18.18%	90.03	0.117	45.45%	85.86	0.164	36.36%
Area	1	53.3	0	0.00%	99.4	0	0.00%	53.6	0	0.00%
	2	79.59	0.158	14.71%	91.12	0.113	44.12%	86.95	0.12	23.53%
	3	70.76	0.248	22.22%	86.4	0.123	22.22%	81.58	0.245	22.22%
Source	1	53.3	0	0.00%	99.4	0	0.00%	53.6	0	0.00%
	2	82.97	0.142	23.53%	90.59	0.111	35.29%	91.5	0.1	35%
	3	74.32	0.198	11.54%	89.83	0.12	53.85%	82.12	0.171	15.38%
Summary statistics		70.25	0.188	11.29	92.97	0.105	27.42	75.95	0.196	16.13

Abbreviations: SD = Standard Deviation; TE = Technical Efficiency.

### 3.3. Tobit Analysis Results

The results of the Tobit analysis found several statistically significant predictors of TE, PTE, and SE scores (Table 4). The staff-to-bed ratio (*p* = 0.011) was the only variable that was significantly associated with the CRSTE scores. Regarding VRSTE (PTE), the date (*p* = 0.012) was significantly associated with correspondent DEA scores. There were two other statistically significant relationships to the SE scores: the type of institution to which an EMRRT belonged (*p* = 0.007) and the staff-to-bed ratio (*p* = 0.043).

**Table 4:** Tobit analysis using CRSTE, VRSTE, SE as the dependent variables.

Variables	CRSTE				VRSTE				Scale Efficiency			
	Coef.	SD	P	95% CI	Coef.	SD	P	95% CI	Coef.	SD	P	95% CI
date	−0.036	0.020	0.085	−0.076–0.005	−0.051	0.019	0.012	−0.089–0.012	0.014	0.020	0.476	0.054–0.026
area	0.026	0.058	0.654	0.142–0.090	−0.052	0.052	0.318	−0.156–0.052	0.120	0.069	0.087	0.018–0.259
Source	−0.026	0.038	0.495	−0.103–0.05	0.060	0.036	0.100	0.012–0.130	−0.128	0.046	0.007	−0.220–0.036
S-B Ratio	0.116	0.044	0.011	0.204–0.028	0.077	0.041	0.064	0.005–0.158	0.088	0.042	0.043	0.003–0.173
D-N Ratio	0.002	0.020	0.939	0.042–0.039	−0.014	0.018	0.462	−0.050–0.023	0.022	0.020	0.281	0.018–0.062

Abbreviations: Coef. = Coefficient; SD = Standard Deviation; 95% CI, 95% Conf. Interval.

## 4. Discussion 

Extant studies on aspects of medical rescue activities for earthquake relief have yet to assess the efficiency of medical rescue efforts after an earthquake. Moreover, there is a lack of consensus on the definition of efficiency of rescue teams and on how to conduct an objective evaluation. This study is the first to use EMRRTs as DMUs and evaluate the technical efficiency of EMRRTs using the DEA method from the micro perspective. Based on EMRRTs’ primary rescue mission mandate and the input and output variables used in the DEA model of health services in non-emergencies, three inputs and two outputs were identified. The results of the analysis found that 38.71% of the units’ PTE reached 1.0 and the average CRSTE, VRSTE, and SE scores were 77.95%, 89.00%, and 87.47%, respectively. These results are generally consistent with those of previous studies, which found that rescue was swift and effective [6,19].

Many previous studies have analyzed the changes to total factor productivity over time using the Malmquist productivity index (MPI). MPI measures output changes resulting from the levels of inputs between two points in time. The values indicate the shifts in productivity of each production unit relative to the observed frontier [18,20]. However, in this study, the observation period was so short (five days) that it can be considered as one period, namely, the emergency period of earthquake relief. Moreover, the dates that the 15 EMRRTs arrived on scene and started working differed, accounting for the difference between the 62 units analyzed and the 75 theoretical units. Specifically, seven teams started on the day of the earthquake, three started on the first day after the earthquake, and five started on the second day after the earthquake. These different dates and the associated rescue data can exactly reflect the factual emergency medical rescue situation. Therefore, to maximize the retention and use of these data, this study used data on the daily rescue operations of these 15 EMRRTs during the study-defined emergency period as a single DMU and the date was employed as a variable that could possibly influence efficiency.

The results of the Tobit regression analysis found that an earlier rescue date was associated with higher PTE. In other words, the daily PTE after the earthquake gradually declined over time. Apparently, the so-called golden period for treating sick and injured people corresponds to a so-called golden period of EMRRTs’ PTE. The decline in PTE may be related to increased rescue workload over time and/or increased worker fatigue. The results further suggest that the sooner rescue efforts reach disaster areas, the better is the rescue efficiency, which points to the need to strengthen the rapid response capacities and mobility of EMRRTs [21,22].

Another important factor to TE was staff-to-bed ratio. In previous studies on health services, staff-to-bed ratio has been a common indicator of a doctor’s workload [23]. This study applies it for the first time in an analysis of disaster relief and humanitarian assistance. The results found that, when in the range of 1.38–3.50, staff-to-bed ratio was positively related to EMRRTs’ TE.

The Chinese government’s approach to Yushu earthquake relief was to evacuate all of the critically injured people outside the disaster area for treatment [8,19,24]. As a result, the first responders primarily provided first-aid treatment and emergency surgical care to the injured people, who were then sent to the evacuation assembly point, where they awaited transport to rearward hospitals. Therefore, there were fewer injured people needing beds at the front lines of the disaster areas. Furthermore, medical teams were usually deployed near the victim assembly points. They had a regular routine, during which they checked on the status of and treated patients at the victim assembly points. Therefore, there were excess (empty) beds at the disaster locations, suggesting that the allocation of medical resources should be flexible and specific to the type of rescue operation and to the unified decision-making system. Robertson *et al.* proposed that small forward teams should be deployed in the early phase of a health disaster response [5]. One of the important tasks of these forward teams would be early and rapid assessment of the needs of the disaster-affected areas to facilitate effective allocation of resources to meet immediate needs and resources to needs assessments to mitigate further adverse health effects [25,26].

The EMRRTs in the emergency medical rescue efforts after the Yushu earthquake included: (1) 42 medical teams from outside Qinghai Province, mainly from Sichuan, Shaanxi, Gansu, and other neighboring provinces and cities; (2) 27 medical teams from Qinghai Province; and (3) 502 local (Yushu) healthcare personnel who participated in medical rescue efforts. In the present study, no difference was found in EMRRT efficiency among these three types of rescuers. This result is consistent with the response efforts to the Wenchuan earthquake [7], Hurricane Katrina [27], and the Haitian earthquake [28], where supporting teams were as important as local forces. Previous studies have proposed that the proximate rescue teams should be deployed because speedy arrival on the scene is good for life-saving rescue time and small regional differences are conducive to medical relief efforts [29]. It was reported that, in the Yushu earthquake relief efforts, because the disaster area was on a high plateau, a medical team from the plains area of Guangdong Province had to evacuate because it experienced a high incidence of altitude sickness when it arrived on the scene [30].

Among the emergency rescue teams that responded to the Yushu disaster, some were from comprehensive tertiary hospitals that had strong technological advantages and some were from primary hospitals, such as county health centers. In this study, the type of institution to which an EMRRT belonged had no significant effect on the TE or PTE of the EMRRT. However, the type of institution was significantly related to the SE. The results of the telephone interview survey found that, during the emergency rescue period, the primary task of all on-site medical teams was to carry out on-site first-aid treatment and to speedily evacuate the victims after the badly injured people were stabilized. Three days after the earthquake, the number of injured people had been reduced and many patients, with respiratory diseases, digestive diseases, and skin diseases who would be treated at local medical institutions under ordinary circumstances, were being admitted to EMRRTs because the local healthcare centers had been destroyed by the earthquake. Therefore, EMRRTs from tertiary hospitals did not demonstrate significant technological advantages over those from primary hospitals simply because technology was not a factor relevant to treatment. On the other hand, the EMRRTs from primary hospitals (with an average staff of 10.25 workers) had a significantly smaller volume than EMRRTs from tertiary hospitals (with an average staff of 16.93 workers)**,** although primary EMRRTs had higher SE. This result is consistent with the results of previous studies that suggested medical teams should be divided into small groups for rescue work during the emergency rescue period [31].

Last, the results found that the doctor-to-nurse ratio was not significantly related to the TE of EMRRTs. The staff structure of Disaster Medical Assistance Teams in the US is relatively equal across staff positions at 35 doctors, nurses, physician assistants, nurse practitioners, pharmacists, paramedics, and logistics and communications staff [4,32]. In 2010, China began establishing 22 health emergency teams throughout the country that would respond to a variety of health emergencies [2]. However, the exact sizes and staffing of these teams has not been clearly defined and the determination of the optimal structure needs further investigation by future studies.

The current study has limitations that are worth noting. In our investigation, most medical rescue records of EMRRTs did not include all of the data on the relevant variables due to the chaotic disaster conditions, resulting in the problem of missing data. As a result, although we investigated many EMRRTs, only 15 of them had complete records and were, therefore, included in the study. The problems related to chaos that yield incomplete or inaccurate reporting need to be addressed, and ways to manage the available data to allow for optimal use without degrading the quality, particularly regarding information about the rescue processes for patients wounded in earthquakes, should be solved in the near future.

## 5. Conclusions 

In conclusion, this study assessed the effectiveness of first responder medical response to the 2010 Yushu, China, earthquake in the first five days after the disaster. It aimed to statistically understand the biggest problems, low rescue efficiency and inefficient resource management, in disaster emergency rescue. The study also addressed the possible influences on effectiveness of other variables (staff-to-bed ratio, rescue date, type of institution to which an EMRRT belonged, and so on), which provides evidence that supports improvements to EMRRT efficiency and a reference for emergency first medical assistance in earthquake relief.
